# The antimalarial efficacy and mechanism of resistance of the novel chemotype DDD01034957

**DOI:** 10.1038/s41598-021-81343-z

**Published:** 2021-01-21

**Authors:** Celia Miguel-Blanco, James M. Murithi, Ernest Diez Benavente, Fiona Angrisano, Katarzyna A. Sala, Donelly A. van Schalkwyk, Manu Vanaerschot, Frank Schwach, Matthew J. Fuchter, Oliver Billker, Colin J. Sutherland, Susana G. Campino, Taane G. Clark, Andrew M. Blagborough, David A. Fidock, Esperanza Herreros, Francisco Javier Gamo, Jake Baum, Michael J. Delves

**Affiliations:** 1 Global Health, GlaxoSmithKline, Tres Cantos, 28760 Madrid, Spain; 2 Department of Microbiology and Immunology, Columbia University Irving Medical Center, New York, NY 10032 USA; 3 Department of Infection Biology, Faculty of Infectious and Tropical Diseases, London School of Hygiene and Tropical Medicine, London, WC1E 7HT UK; 4 Division of Microbiology and Parasitology, Department of Pathology, Cambridge University, Tennis Court Road, Cambridge, CB2 1QP UK; 5 Department of Life Sciences, Imperial College London, South Kensington, London, SW7 2AZ UK; 6 Parasites and Microbes Programme, Wellcome Trust Sanger Institute, Hinxton, CB10 1SA UK; 7 Department of Chemistry, Molecular Sciences Research Hub, Imperial College London, White City Campus, Wood Lane, London, W12 OBZ UK; 8 Department of Molecular Biology, The Laboratory for Molecular Infection Medicine Sweden (MIMS), Umeå University, 901 87 Umeå, Sweden; 9 Division of Infectious Diseases, Department of Medicine, Columbia University Irving Medical Center, New York, NY 10032 USA; 10 Present Address: Medicines for Malaria Venture, 20 Route de Pré-Bois, 1215 Geneva 15, Switzerland

**Keywords:** Malaria, Drug discovery and development

## Abstract

New antimalarial therapeutics are needed to ensure that malaria cases continue to be driven down, as both emerging parasite resistance to frontline chemotherapies and mosquito resistance to current insecticides threaten control programmes. *Plasmodium*, the apicomplexan parasite responsible for malaria, causes disease pathology through repeated cycles of invasion and replication within host erythrocytes (the asexual cycle). Antimalarial drugs primarily target this cycle, seeking to reduce parasite burden within the host as fast as possible and to supress recrudescence for as long as possible. Intense phenotypic drug screening efforts have identified a number of promising new antimalarial molecules. Particularly important is the identification of compounds with new modes of action within the parasite to combat existing drug resistance and suitable for formulation of efficacious combination therapies. Here we detail the antimalarial properties of DDD01034957—a novel antimalarial molecule which is fast-acting and potent against drug resistant strains in vitro, shows activity in vivo, and possesses a resistance mechanism linked to the membrane transporter Pf*ABCI3*. These data support further medicinal chemistry lead-optimization of DDD01034957 as a novel antimalarial chemical class and provide new insights to further reduce in vivo metabolic clearance.

## Introduction

Malaria, caused by *Plasmodium* parasites, remains a global disease of devastating morbidity and mortality. Despite recent successes in malaria control and eradication in the last two decades, progress has stalled, with an estimated 228 million cases and 405,000 deaths in 2018^1^. Together with the emergence of artemisinin and partner drug resistance in Southeast Asia, there is a pressing need for new chemical classes of antimalarials with unique modes of action. In particular, molecules that are fast-acting and long-lasting are a priority for antimalarial development as they have the potential to rapidly reduce the parasite burden within an afflicted patient and provide protection against recrudescence (Target Candidate Profile 1)^2^.

**Figure 1:**
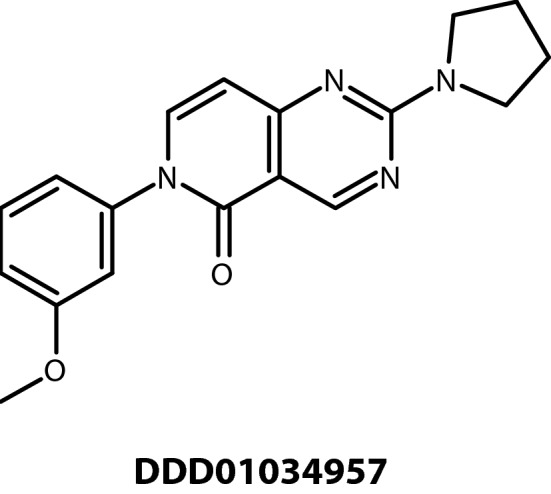
The chemical structure of DDD01034957 that was identified in a previous screen against *P. falciparum* asexual development and reportedly showed an IC_50_ of 172nM^4^.

Recently, *P. falciparum* asexual stages and male and female gametocytes were screened against a 70,000 compound diversity library identifying a number of new scaffolds for antimalarial drug development^3^. One such compound, DDD01034957 (Fig. 1), was identified to possess a chemical scaffold not represented in previous high throughput antimalarial screens. As a novel chemotype, together with its with its reported 172 nM IC_50_ against *P. falciparum* asexual parasites (but not gametocytes), this compound was identified as a promising hit molecule and therefore prioritised for further study.

Here we perform a detailed in vitro and in vivo characterisation of the antimalarial and pharmacokinetic properties of DDD01034957 using three different *Plasmodium* species, test for development of resistance in vitro and perform structure–function studies of analogues which share the same chemical scaffold.

## Results

### DDD01034957 shows no cross-resistance to a range of drug resistant *P. falciparum* strains

The use of partner drugs in combination therapies requires two molecules with distinct modes of action to protect against the emergence of resistance to the monotherapy. To determine whether DDD01034957 has a unique mode of action or acts through an already recognised mechanism, its efficacy was tested in asexual growth assays against a range of selection-derived drug resistant *P. falciparum* parasites and their parental non-resistant lines (Table 1). In all assays, the resistant strains were sensitive to DDD01034957, giving no greater than a threefold increase in IC_50_ when compared to their parental lines. This result suggests that DDD01034957 has a distinct mode of resistance and is active against parasites harbouring resistance mutations linked to a loss of efficacy against many new antimalarials currently under clinical development.

**Table 1:** DDD01034957 is efficacious against common resistant parasite lines.

Target/pathway	Parent line	Mutant/transformed strain	DDD01034957 fold change in IC_50_
PfATP4	3D7	3D7-C9-ATP4F917L^5^	0.83 ± 0.03
	W2	W2-ATP4P412L^5^	1.65 ± 0.20
	W2	W2-ATP4V178I^6^	1.47 ± 0.35
PfCARL	3D7	3D7-C9-CARL-E834D^7^	0.83 ± 0.11
	3D7	3D7-C9-CARL-Q821H^7^	1.16 ± 0.13
PfPI4K	Dd2	Dd2-PI4K-A1319V^8^	1.10 ± 0.08
ETC	Dd2	Dd2attB_yeastDHODH^9^	0.90 ± 0.06
Folate Biosynthesis	3D7	3D7 adapted to grow in ↓ pABA/FA^10^	2.96 ± 0.29

DDD01034957 was tested against a range of published drug resistant parasite strains and their parental lines in asexual growth assays with parasite lactate dehydrogenase activity (pLDH) as a readout for parasite growth. Presented data is the mean fold change in IC_50_ of three to nine independent replicates ± SEM.

### DDD01034957 is a fast-acting antimalarial against *P. falciparum* asexual stages in vitro

**Figure 2:**
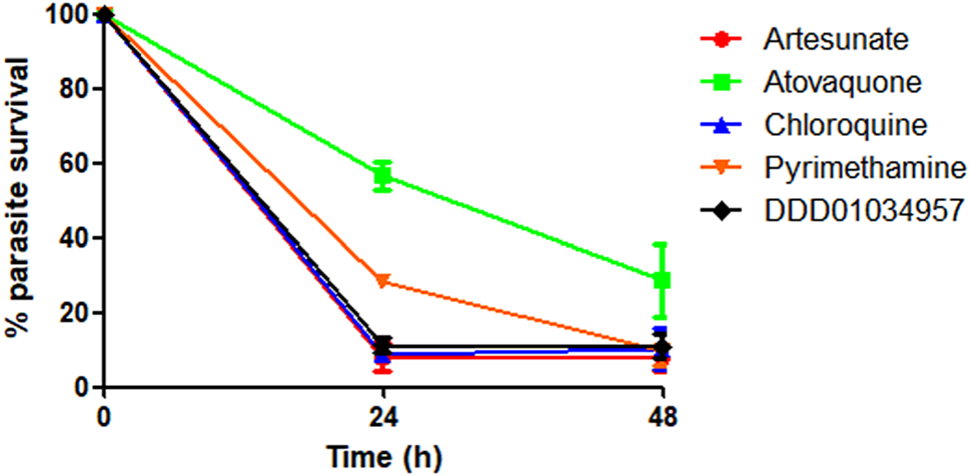
DDD1034957 rapidly kills P. falciparum 3D7 strain asexual parasites. Parasites were treated either with ten-times (10 ×) the asexual IC_50_ of DDD1034957 or 10 × IC_50_ of a range of control antimalarials and then sampled at 24 h and 48 h after treatment. Samples were washed to remove drugs and cultured with fresh erythrocytes that had been fluorescently labelled with carboxyfluorescein diacetate succinimidyl ester (CFDA-SE). After a further 48 h, parasites were labelled with Hoechst 33342 and these doubly-labelled cells (indicating viable parasites that had invaded fresh erythrocytes after drug treatment) were quantified by flow cytometry. DDD1034957 achieved rapid parasite kill after only 24 h treatment showing fast-acting kinetics similar to artesunate and chloroquine. Data shown is the mean of three independent experiments and error bars denote the SD.

Fast-acting antimalarials are crucial to rapidly reduce the parasite burden and relieve the patient from malarial symptoms as fast as possible^11^. To determine the speed of action of DDD01034957, it was evaluated in an established parasite viability assay^12^ and compared to artesunate (fast kill), chloroquine (fast kill), pyrimethamine (medium kill) and atovaquone (slow kill)^12^. At 3.2 µM (10xIC_50_), DDD01034957 rapidly reduced parasite viability to baseline within 24 h at a similar rate to 10xIC_50_ artesunate and chloroquine (Fig. 2), thus demonstrating its fast-acting antimalarial activity. In contrast, the slower acting antimalarials pyrimethamine and atovaquone showed only partial reduction in parasite viability at 24 h.

### In vitro selection of *P. falciparum* clones resistant to DDD01034957 identifies *pfabci3* as a mediator of resistance

**Figure 3:**
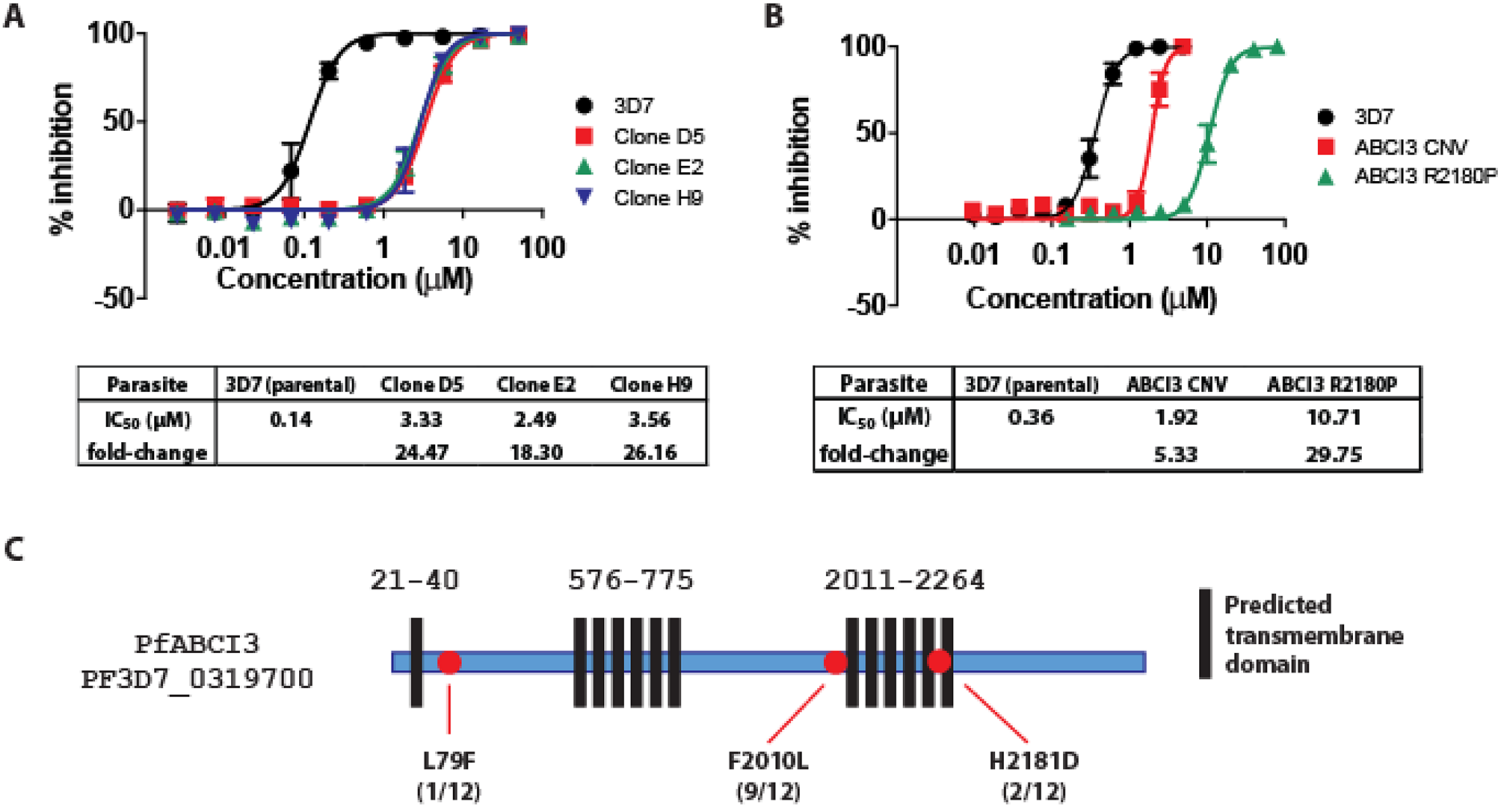
The generation and genotyping of parasite resistance to DDD01034957. (**A**) After selection pressure, DDD01034957 was less efficacious against drug-resistant clonal parasite lines compared to the parental 3D7 line. The graph illustrates a shift in efficacy of three representative clones. Error bars denote the SEM based upon three independent repeats. (**B**) DDD01034957 was also less efficacious against a pfabci3 copy number variant parasite line and a mutant line harbouring the pfabci3 R2180P mutation. Error bars denote the SEM based upon at least three independent replicates. (**C**) Whole-genome sequencing of twelve clones from the selection experiments revealed that the majority had a single point mutation mapping to the predicted second multi-transmembrane region of PfABCI3 with one clone harbouring a mutation towards the N terminal end of the protein. Protein transmembrane predictions calculated by PlasmoDB (https://plasmodb.org/plasmo/).

To identify the mode of action of DDD01034957 and/or identify a mechanism of resistance, drug resistant parasites were selected in vitro by pulsing *P. falciparum* asexual blood stage cultures with DDD01034957 at 0.2 µM and 2 µM. Approximately three weeks later, the resistant parasite population were serially diluted to derive clonal parasite lines with distinct genotypes. Eleven clones from three independent selection experiments were isolated and, of the nine tested, all showed a 11.5- to 26.2-fold increase in IC_50_ (Fig. 3A, Supplementary Table 1). Whole-genome sequencing highlighted two genes in common that possessed a variety of single point mutations in all eleven clones—*pfabci3* (PF3D7_0319700) and *pflsa1* (PF3D7_10364000). Although each clone possessed up to two different mutations in *pflsa1* from a combination of six mutations observed, it was discounted as a potential mode of action/mechanism of resistance due to the reported exclusive expression of its gene product, liver stage antigen 1, at only the liver stages of the parasite life cycle^13^. The remaining gene *pfabci3* encodes an essential ATP-binding cassette transport protein that is known to mediate resistance to other experimental antimalarial compounds. These compounds encompass several distinct chemical scaffolds^14,15^ unrelated to DDD01034957. Eight out of eleven clones harboured the mutation F2010L, two possessed a H2181D mutation and the remaining clone acquired a L79F mutation. The F2010L and H2181D mutations map to a region of the ABCI3 protein that is predicted to harbour multiple transmembrane domains which may form part of the transporter channel (Fig. 3C). To determine whether DDD01034957 resistance is mediated by PfABCI3, this compound was tested against a previously reported line harbouring a R2180P mutation (the amino acid adjacent to the H2181D mutation observed in this study) and a *pfabci3* copy number variant (CNV) (Fig. 3B)^15^. The R2180P mutant was 29.8-fold less susceptible to DDD01034957 than the parental strain, while the CNV line displayed 5.3-fold decreased susceptibility. Together, these data strongly suggest that PfABCI3 does indeed mediate resistance to DDD01034957.

A combined dataset of 6,230 sequenced *P. falciparum* isolates from 22 countries^16–18^ was searched and none of the identified mutations in *pfabci3* conferring DDD01034957 resistance were found. Similarly, evidence of *pfabci3* CNV was only found in 0.89% of isolates, which possessed duplications of different lengths implying independent events rather than selection pressure at this locus. This implies that an optimised scaffold of DDD01034957 would not encounter existing Pf*ABCI3*-mediated resistance in the clinic.

### Determining the in vivo efficacy and pharmacokinetic properties of DDD01034957

**Figure 4:**
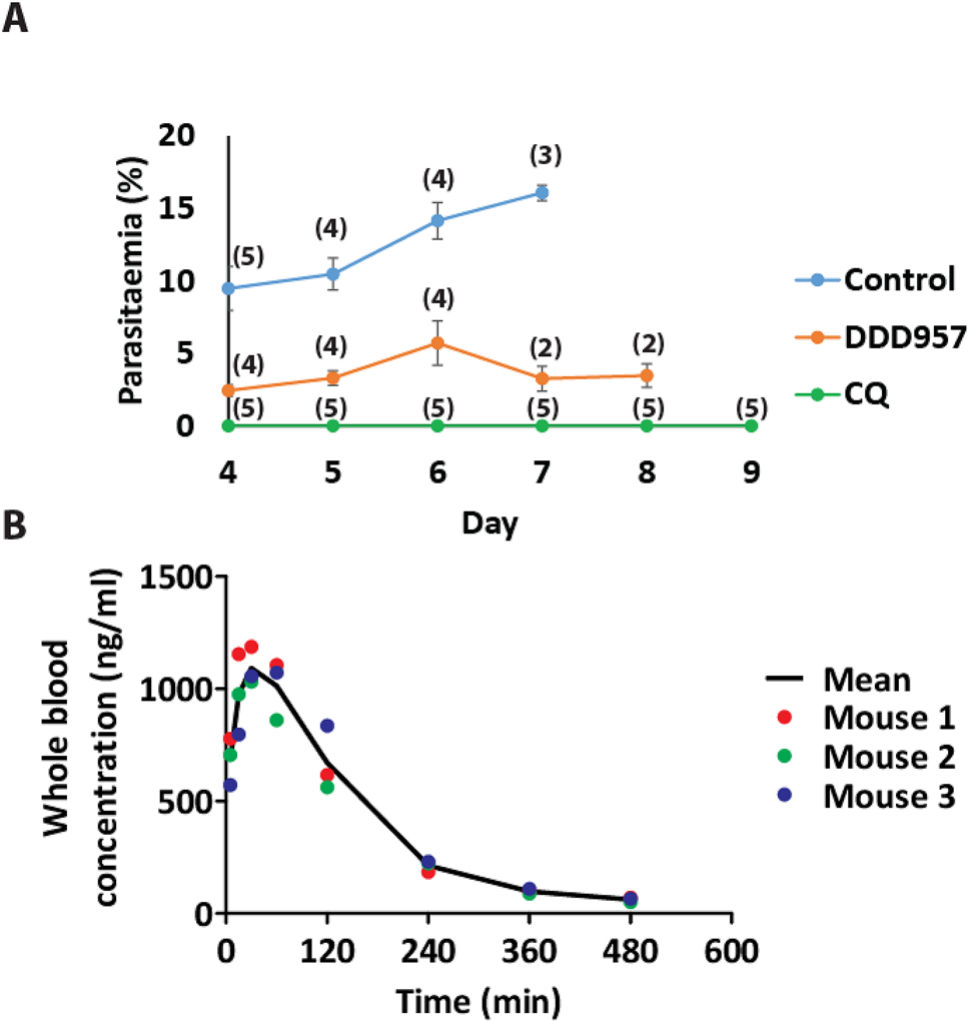
In vivo efficacy of DDD01034957 in rodent malaria and pharmacokinetic properties in mice. (**A**) Plasmodium berghei infected mice were treated orally with 50 mg/kg DDD01034957 or 10 mg/kg chloroquine in the 4-day suppression test. DDD01034957 reduced parasitaemia in infected mice whilst chloroquine completely supressed infection. Numbers in brackets indicate the number of mice remaining in the cohort on a particular day (i.e. those that had not reached their humane endpoint). A treatment cohort was ceased when all mice in the cohort had reached their humane endpoint or after 20 days if no malaria parasitaemia or symptoms were observed. Error bars denote SEM. (**B**) Female BALB/c mice (n = 3) were treated with 50 mg/kg DDD01034957 by intraperitoneal injection and whole blood concentration sampled over time.

Having established in vitro antimalarial efficacy, the in vivo efficacy of DDD01034957 was investigated using the standard 4-day suppression test in the *Plasmodium berghei* rodent model of infection^19^. A 50 mg/kg oral dose of DDD01034957 administered on four consecutive days to infected mice reduced parasitaemia by 59.7–79.8% compared to the vehicle control (Fig. 4A). However, at this level of suppression DDD01034957 did not appear to protect mice from the symptoms of malaria and there was no statistically significant difference in time taken for mice to reach their humane endpoint (unpaired t-test). In contrast, the 10 mg/kg chloroquine treatment completely supressed parasitaemia and all mice survived to the end of the experiment. Whole blood levels of DDD01034957 in naïve mice treated with a 50 mg/kg dose by intraperitoneal (IP) injection rapidly peaked at 1092 ng/ml but reduced with an elimination half-life of ~ 106 min suggesting a rapid clearance mechanism (Fig. 4B).

### In vitro antimalarial structure–activity relationship

**Figure 5:**
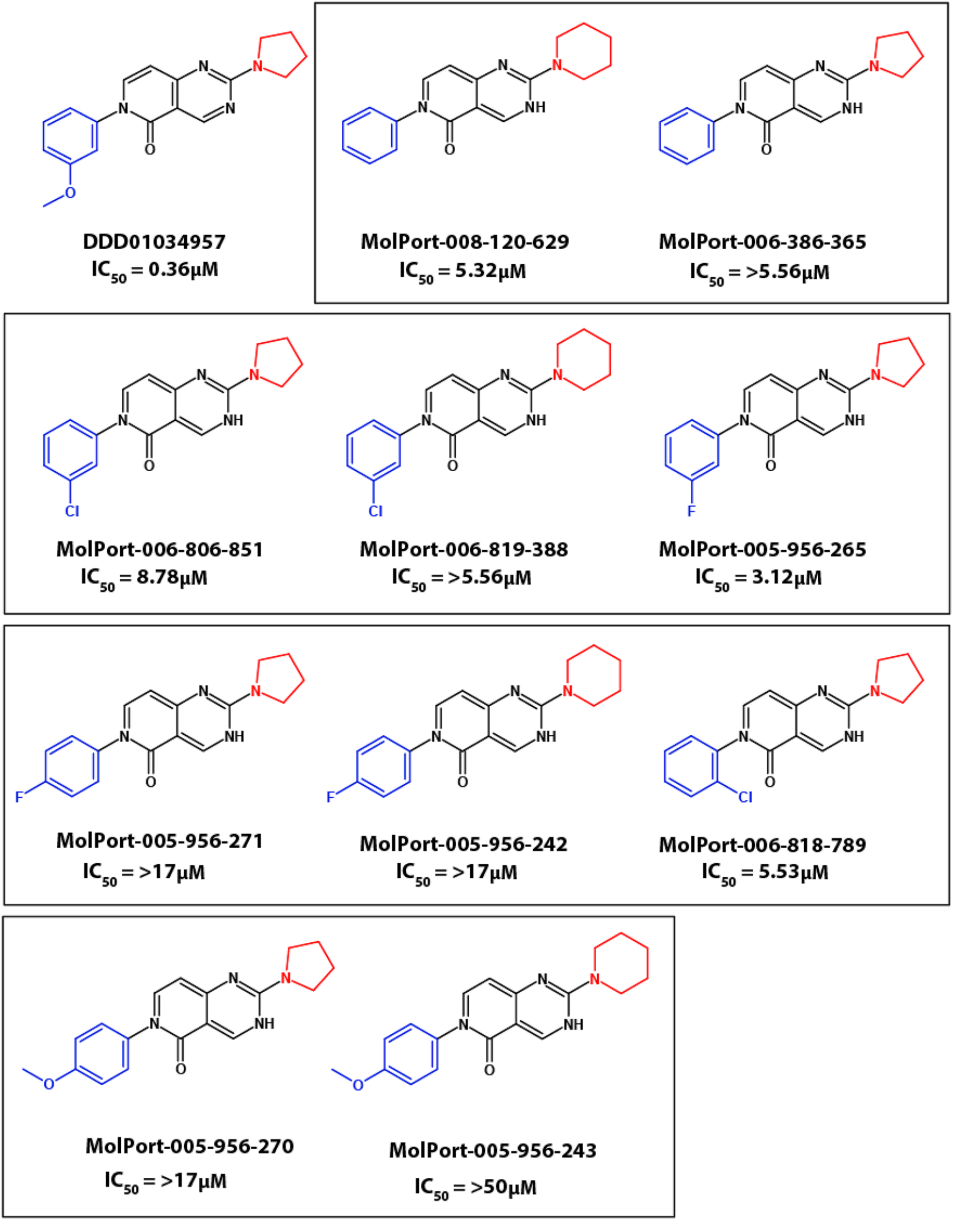
Structure–activity relationship of DDD01034957. Ten commercially available analogues of DDD01034957 were tested for antimalarial activity in the P. falciparum asexual growth assay and efficacy compared to the parent molecule.

It was hypothesised that the methoxy group on the phenyl ring of DDD01034957 could be a potential metabolic liability and partly account for its fast in vivo clearance following metabolic cleavage of the methyl group. Therefore, commercially available analogues in which the methoxy group was shifted, deleted, or replaced with polar halogens in a variety of positions were purchased and tested for in vitro activity against *P. falciparum* asexual parasites (Fig. 5). Despite having the same number of hydrogen bond donors and acceptors, moving the methoxy group from the meta to para position (MolPort-005-956-270 and MolPort-005-956-243) completely abrogated antimalarial activity. Similarly, halogen substitution of the para-methoxy group was not active (Molport-005-956-271 and Molport-005-956-242), whilst the meta (Molport-006-806-851, MolPort-006-819-388 and Molport-005-956-265) and ortho (Molport-006-818-789) substitutions retained low micromolar activity. Removing the methoxy group altogether also greatly reduced antimalarial activity (MolPort-008-120-629 and MolPort-006-386-365) suggesting that the methoxy group of DDD01034957 may be important for target binding. Conversely, replacement of the five-membered amine ring with a six-membered ring did not appreciably modify activity suggesting that at least small changes of this part of the molecule are tolerated, and further alterations will need to be investigated.

### DDD01034957 is more efficacious against *P. falciparum* than *P. knowlesi*

**Figure 6:**
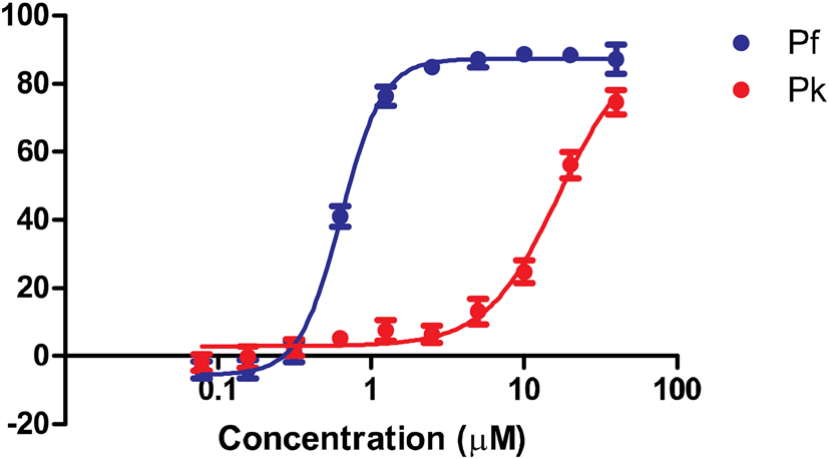
A comparison of the in vitro asexual efficacy of DDD01034957 against P. falciparum (Pf) and P. knowlesi (Pk) over one asexual cycle. Dose response data shows the mean of eight independent replicates with error bars denoting the SEM.

It has been established that some antimalarials show different efficacy against different species of *Plasmodium*^20^. Therefore, the antimalarial activity of DDD01034957 was tested on culture adapted *Plasmodium knowlesi*—a species causing malaria in humans and primates common in Southeast Asia. Over one asexual cycle (48 h *P. falciparum* vs. 27 h *P. knowlesi*), DDD01034957 showed greatly reduced potency against *P. knowlesi* with inhibition not reaching an upper plateau by the maximum concentration tested (40 µM) (Fig. 6).

## Discussion

Herein, we report the in vitro and in vivo efficacy of DDD01034957 together with its mechanism of resistance mediated through the transporter PfABCI3. DDD01034957 demonstrates promising fast-acting killing of *P. falciparum* asexual blood stage parasites in vitro, and shows efficacy against parasite lines harbouring resistance to many diverse antimalarials. These data support the continued development of DDD01034957 as a new chemotype with antimalarial potential. However, DDD01034957 had limited in vivo efficacy against *P. berghei*. Whole blood concentrations peaked at 25-times the in vitro IC_50_ and maintained levels of DDD01034957 above the IC_50_ for at least 480 min (the last time point sampled), indicating that lack of efficacy may not be due to bioavailability, but in part may be related to fast clearance. Interestingly however, the *pbabci3* gene harbours the equivalent histidine to aspartate point mutation that gave the ~ 12-fold increase in IC_50_ in the *P. falciparum* H2181D resistant line, suggesting that the *P. berghei* line tested may be naturally resistant to DDD01034957. Consequently, future work should prioritise studying in vivo efficacy of DDD01034957 in the humanised *P. falciparum* mouse model or alternatively in a *P. berghei* line modified transgenetically to contain the D2181H reverting mutation. DDD01034957 lacked efficacy against *P. knowlesi* which does not contain any of the point mutations in *pkabci3* linked to resistance in *P. falciparum*. It has previously been reported that *P. knowlesi* shows modulated sensitivity to a range of different antimalarials when compared to *P. falciparum* ^20^. Currently it is unclear as to why this phenomenon occurs. However *P. falciparum* possesses a number of species-specific influx/efflux transporters that might play an important role in modulating the uptake of DDD0103957 from the red blood cell^21^. Alternatively, DDD010349757 efficacy could be linked to different binding affinities to its target, which might differ between *Plasmodium* species.

A preliminary structure–activity relationship (SAR) study using commercially available analogues indicates that modification of the methoxy group of DDD01034957 substantially impacted its antimalarial activity. Replacement with a fluorine or chlorine atom results in a reduced activity by 10- to 14-fold respectively. Deeper medicinal chemistry studies are required to determine whether metabolism of the methoxy group to a phenol is important for in vivo clearance. Key to such studies could be the synthesis and testing of phenol analogues of our series and/or methoxy surrogates. Additional future medicinal chemistry could include exploration of whether modification of the pyrido-pyrimidinone core can increase efficacy, while improving pharmacokinetics.

In conclusion, a detailed study of the antimalarial efficacy of new chemotypes is highly valuable for the progression of molecules into the lead optimisation stage. DDD01034957 shows promising in vitro activity and demonstrated, albeit reduced, oral in vivo efficacy (which is unsurprising for a non-optimised primary hit at this preliminary stage). Its mode of action is potentially distinct from other antimalarials under development and its mechanism of resistance is now defined, implicating the transporter PfABCI3 in drug influx/efflux. Future work will focus on reducing in vivo metabolic clearance, further improving efficacy, and mitigating PfABCI3-mediated resistance.

## Materials and methods

### Comparison of resistant strains

The asexual activity of published drug resistant strains (Table 1) were tested in the *P. falciparum* lactate dehydrogenase assay (pLDH)^3^ which is a surrogate for asexual parasite growth. Each strain was tested in comparison to its parental (non-resistant) line. Parasites were prepared at 0.25% parasitaemia/2% haematocrit in RPMI-1640 supplemented with 5% Albumax and 150 µM hypoxanthine (except the parasite strain adapted to low folate conditions which was cultured in culture medium depleted of para-aminobenzoic acid and with low (100 ng/ml) folic acid concentrations) before being dispensed into wells of a 384 well plate (25 µl per well) containing test compounds in DMSO. After incubation for 72 h at 37 °C under a 5% CO_2_/5% O_2_/90% N_2_ atmosphere, the plates were frozen at − 70 °C to lyse cells. After thawing, 70 µl of reaction solution (100 mM sodium l-lactate, 100 mM 3-acetylpyridine adenine dinucleotide (APAD), 125 µM NitroBlue tetrazolium (NBT), 200 µl ml^−1^ diaphorase, 0.5% tween 20, 100 mM Tris–HCl pH 8) were added to each well and mixed. pLDH activity was then measured by monitoring NBT reduction by absorbance at 650 nm using a plate reader.

### Speed of kill assay

This assay was performed as described in Linares et al.^22^. Briefly, *P. falciparum* 3D7 strain parasites were incubated at 2% haematocrit, 0.5% parasitaemia in 96 well plates in the presence of test compounds at 10xIC_50_. At 24 and 48 h post drug treatment parasite samples were transferred and diluted into fresh erythrocytes labelled with 10 µM of the fluorophore carboxyfluorescein diacetate succinimidyl ester (CFDA-SE) and incubated for a subsequent 48 h. Parasites were then labelled with 2 µM Hoechst 33342 and the proportion of viable parasites that had reinvaded the fresh erythrocytes (i.e. CFDA-SE-positive erythrocytes containing Hoechst 33342-positive parasites) after 24/48 h drug pulse determined by FACS analysis.

### Evolution and testing of drug resistance

A *P. falciparum* line showing an accelerated 38 h asexual life cycle (3D7 IG06—a gift from Daniel Goldberg, Washington University School of Medicine in St Louis) was used for drug selection experiments. Selection of compound-resistant lines was performed using a high-pressure intermittent method^6^. 1 × 10^9^ asexual parasites were cultured in the presence of 0.2 µM or 2 µM DDD01034957 and monitored regularly until parasitaemia recrudesced (11–15 days). Then, compound pressure was removed to allow cultures to expand up to ~ 2% parasitaemia, DDD01034957 was added again and sensitivity was compared to the parental line. Resistant parasites were then cloned by limiting dilution to obtain up to six clones per selection experiment (n = 3).

The sensitivity of a Pf*ABCI3* copy number variant (CNV) and Pf*ABCI3* R2180P^14,15^ mutant to DDD01034957 was tested in asexual growth assays. Parasites were maintained at 3% haematocrit with human O + red blood cells in RPMI medium supplemented with 50 μM hypoxanthine, 2 g/l sodium bicarbonate, 2 mM l-glutamine, 25 mM HEPES, 0.5% AlbuMAXII (Invitrogen) and 10 μg/ml gentamycin in 5% O_2_, 5% CO_2_ and 90% N_2_ at 37** °C**. Parasites were diluted to 0.3% parasitaemia/1% haematocrit in 96 well plates containing serial dilutions of DDD01034957 and incubated for 72 h at 37 °C. Parasite survival in each well was assessed by SYBR Green and MitoTracker Deep Red FM staining (Life Technologies) and subsequent flow-cytometric analysis (Accuri C6, BD Biosciences).

### Whole genome sequencing

Resistant parasite cultures were pelleted by centrifugation and then frozen at − 80 °C to lyse the cells. Genomic DNA was then extracted using a Blood & Cell Culture DNA Mini Kit (Qiagen) and sequenced together on one lane of an Illumina HiSeqX machine with a 150 bp paired end protocol. The resulting sequences were mapped to the 3D7 reference genome (v3) using *bwa-mem* software, and genomic variants called using *GATK* and *samtools* software tools within existing bioinformatic pipelines^23^. A collection of genomes from 6230 *P. falciparum* isolates from 22 countries ^16–18^, was searched for identified Pf*ABCI3* mutations. The same dataset was analysed for evidence of CNV using Delly^24^ with a < 200kbp cut-off.

### *P. berghei* 4-day suppression test

All procedures were performed in accordance with the UK Animals (Scientific Procedures) Act (PPL 70/8788) and approved by the Imperial College and University of Cambridge AWERB. The Office of Laboratory Animal Welfare Assurance for Imperial College covers all Public Health Service supported activities involving live vertebrates in the US (no. A5634-01). This study was carried out in compliance with the ARRIVE guidelines (https://arriveguidelines.org/). Naïve T0 mice (n = 5 per treatment) were infected by IP injection of 2 × 10^7^ *P. berghei* parasites obtained from the blood of a donor mouse. DDD01034957 and chloroquine (positive control) were dissolved in 7% Tween 80/3% Ethanol in dH_2_0 and dosed orally to infected mice to result in a 50 mg/kg and 10 mg/kg exposure respectively. Five additional infected mice received a vehicle-only dose as a negative control. Infected mice then received identical doses of drugs/vehicle 24, 48 and 72 h after infection and commencing at 96 h post-infection, were smeared daily to monitor parasitaemia. Mice reached their humane endpoint immediately upon showing outward pathological indictors of malarial infection and were considered cured if this was not reached by day 30 post-infection.

### Mouse in vivo PK analysis

Pharmacokinetic analysis was outsourced commercially to Dundee Drug Discovery Unit (DDU). Briefly, DDD01034957 was dissolved in 7% Tween 80/3% Ethanol in dH_2_0 and 50 mg/kg and administered by IP injection to three BALB/c mice. Whole blood was sampled at intervals over 8 h and the concentration of DDD01034957 determined by mass spectroscopy.

### Analogue asexual growth assays

*P. falciparum* 3D7 strain parasites were synchronised by sorbitol lysis to obtain a pure ring stage population and maintained in RPMI 1640 supplemented with 25 mM HEPES, 25 mM Na_2_HPO_3_, 10 mM d-glucose, 2 mM l-glutamine, 50 mg/L hypoxanthine, 25 mg/L gentamicin sulphate, 5 g/L Albumax II. Parasites were diluted to 2% parasitaemia and 1% haematocrit before being cultured in 96 well plates containing serial dilutions of DDD01034957 and analogue compounds. Wells containing DMSO served as a negative control and wells containing 100 nM dihydroartemisinin served as a positive control. Parasites were maintained at 37 °C under a gas mixture of 96% N_2_, 3% O_2_ and 1% CO_2_ for 72 h before plates were frozen at − 80 °C overnight. Thawed plates were lysed in SYBR green lysis buffer (20 mM Tris, 5 mM EDTA, 0.008% (w/v) saponin, 0.08% (v/v) Triton X-100, pH 7.5 + 1:10,000 SYBR Green I). After 1 h incubation at 37 °C, plates were read in a fluorescence plate reader at 490 nm excitation and 520 nm emission. Percentage inhibition of asexual parasite growth was calculated with respect to the positive and negative controls.

### *P. falciparum* vs. *P. knowlesi* asexual assay comparison

*P. falciparum* 3D7 strain and *P. knowlesi* A1-H.1 strain were maintained in RPMI 1640 supplemented with 25 mM HEPES, 25 mM Na_2_HPO_3_, 10 mM D-glucose, 2 mM L-glutamine, 50 mg/L hypoxanthine, 25 mg/L gentamicin sulphate, 5 g/L Albumax II and 10% (v/v) equine serum (Pan Biotech, P30-0702). Both species were cultured in human A + erythrocytes at 37 °C under a gas mixture of 96% N_2_, 3% O_2_ and 1% CO_2_. Drug susceptibility assays were initiated with unsynchronised parasites diluted to 1% haematocrit and 1% parasitaemia in 96 well plates containing serial dilutions of DDD01034957. Assays were run for one asexual cycle (48 h *P. falciparum*; 27 h *P. knowlesi*) before being frozen at − 80 °C overnight. Thawed plates were lysed in SYBR green lysis buffer and fluorescence read as above.

## Supplementary Information

Supplementary Information
