# Supplementary material for: The antimalarial efficacy and mechanism of resistance of the novel chemotype DDD01034957
Source: Sci Rep. 2021 Jan 21;11:1888. doi: 10.1038/s41598-021-81343-z (PMC7820608; doi:10.1038/s41598-021-81343-z)
Supplement: Supplementary file 1 — Supplementary Information [file 41598_2021_81343_MOESM1_ESM.pdf]

The antimalarial efficacy and mechanism of resistance of the novel chemotype DDD01034957

Celia Miguel-Blanco[1], James M. Murithi[2], Ernest Diez Benavente[3], Fiona Angrisano[4], Katarzyna A. Sala[5], Donelly A. van Schalkwyk[3], Manu Vanaerschot[2], Frank Schwach[6], Matthew J. Fuchter[7], Oliver Billker[6,8], Colin J. Sutherland[3], Susana Campino[3], Taane G. Clark[3], Andrew M. Blagborough[4,5], David A. Fidock[2,9], Esperanza Herreros[1,10], Francisco Javier Gamo[1], Jake Baum[5], Michael J. Delves[3*].

*Correspondence to: michael.delves@lshtm.ac.uk

1. Global Health, GlaxoSmithKline, 28760 Tres Cantos, Madrid, Spain.

2. Department of Microbiology and Immunology, Columbia University Irving Medical Center, New York, NY 10032, USA.

3. Department of Infection Biology, Faculty of Infectious and Tropical Diseases, London School of Hygiene & Tropical Medicine, London WC1E 7HT, UK.

4. Division of Microbiology and Parasitology, Department of Pathology, Cambridge University, Tennis Court Road, Cambridge CB2 1QP, UK.

5. Department of Life Sciences, Imperial College London, South Kensington, London SW7 2AZ, UK.

6. Parasites and Microbes Programme, Wellcome Trust Sanger Institute, Hinxton CB10 1SA, UK.

7. Department of Chemistry, Molecular Sciences Research Hub, Imperial College London, White City Campus, Wood Lane, London W12 OBZ, UK.

8. Department of Molecular Biology, The Laboratory for Molecular Infection Medicine Sweden (MIMS), Department of Molecular Biology, Umeå University, Umeå SE-901 87, Sweden.

9. Division of Infectious Diseases, Department of Medicine, Columbia University Irving Medical Center, New York, NY 10032, USA.

10. Present address: Medicines for Malaria Venture, 20 Route de Pré-Bois, 1215 Geneva 15, Switzerland

Parasite	3D7 (parental)	Clone D5	Clone E2	Clone H9	Clone F3	Clone B2	Clone D2	Clone E11	Clone D3	Clone C11	Clone D3	Clone G8
IC_{50} (µM)	0.14	3.33	2.49	3.56	2.62	1.81	2.77	3.28	2.40	1.57	ND	ND
fold-change		24.47	18.30	26.16	19.25	13.30	20.35	24.10	17.63	11.54		
Pf*ABCI3* mutation		F2010L	F2010L	F2010L	F2010L	F2010L	F2010L	F2010L	F2010L	H2181D	L79F	H2181D
		Expt 1			Expt 2					Expt 3		

Supplementary Table S1 - *In vitro* selection of resistance and genotyping. *P. falciparum* asexual parasites were pulsed with DDD01034957 at 0.2µM or 2µM. When recovered, surviving parasites were cloned and tested for resistance to DDD01034957. From three independent selection experiments, twelve clones were obtained with a 12.1 to 26.2-fold increase in IC50 to DDD01034957. Whole genomic sequencing revealed all clones had mutations in PF3D7_0319700 (Pf*ABCI3*).
